# Functional recovery from chronic writer’s cramp by brain-computer interface rehabilitation: a case report

**DOI:** 10.1186/1471-2202-15-103

**Published:** 2014-09-01

**Authors:** Yasunari Hashimoto, Tetsuo Ota, Masahiko Mukaino, Meigen Liu, Junichi Ushiba

**Affiliations:** Department of Electrical and Electronic Engineering, Kitami Institute of Technology, Hokkaido, Japan; Asahikawa Medical University Hospital, Hokkaido, Japan; Department of Rehabilitation Medicine I, School of Medicine, Fujita Health University, Aichi, Japan; Department of Rehabilitation Medicine, Keio University School of Medicine, Tokyo, Japan; Department of Biosciences and Informatics, Faculty of Science and Technology, Keio University, Kanagawa, Japan

**Keywords:** Neurorehabilitation, Neurofeedback, Upper extremity, Motor learning, Cortico-muscular coherence

## Abstract

**Background:**

Dystonia is often currently treated with botulinum toxin injections to spastic muscles, or deep brain stimulation to the basal ganglia. In addition to these pharmacological or neurosurgical measures, a new noninvasive treatment concept, functional modulation using a brain-computer interface, was tested for feasibility. We recorded electroencephalograms (EEGs) over the bilateral sensorimotor cortex from a patient suffering from chronic writer’s cramp. The patient was asked to suppress an exaggerated beta frequency component in the EEG during hand extension.

**Results:**

The patient completed biweekly one-hour training for 5 months without any adverse effects. Significant decrease of the beta frequency component during handwriting was confirmed, and was associated with clear functional improvement.

**Conclusion:**

The current pilot study suggests that a brain-computer Interface can give explicit feedback of ongoing cortical excitability to patients with dystonia and allow them to suppress exaggerated neural activity, resulting in functional recovery.

## Background

Brain-Computer Interface (BCI) technology has already been successfully used to control a computer mouse cursor and a robotic arm by thought, and thus has been expected to become a tool to compensate for lost motor functions in patients with spinal cord injury [1] or amyotrophic lateral sclerosis [2]. Recently, some research groups succeeded in showing another possible use of BCI, that is, as a tool to promote neural plasticity causing functional recovery from stroke [3–9]. The number of clinical applications of such BCI-based neurorehabilitation is expected to increase in the near future.

Focal dystonia is a disorder of movement characterized by involuntary, sustained muscle contractions, frequently causing twisting and repetitive movements or abnormal postures of a body part [10]. Writer's cramp (WC) is an example of task-specific focal hand dystonia. WC was once believed to be a purely psychological problem, but more recently is understood to be due to more specific neural dysfunction, including that of the basal ganglia [11, 12]. Disinhibition and overexcitation of the cortico-basal ganglia-thalamic loop may lead to co-contractions and dystonic postures [13].

A recent transcranial magnetic stimulation study has also revealed that there is shift in the balance between excitation and inhibition in local circuits of the primary motor cortex in focal dystonia [13]. Sensory evoked potential recordings in focal dystonia (and in other types of dystonic disorders) also indicate that in dystonia there is impaired inhibition at spinal and cortical levels of the somatosensory system, which can lead to an abnormal sensory assistance to ongoing motor programs, resulting in motor abnormalities [14].

Toro et al. investigated abnormal activity at the cortical level with a scalp electroencephalogram [15]. In their results, a significantly lower amount of event-related desynchronization (ERD) in the 20–30 Hz band was found in dystonic patients over the contralateral and midline centroparietal regions, from −100 ms to +200 ms with respect to electromyogram (EMG) onset. If we could provide explicit online representation of such dystonic-specific EEG features, it could help WC patients to voluntarily return the cortical state to normal using BCI neurofeedback.

**Figure 1:**
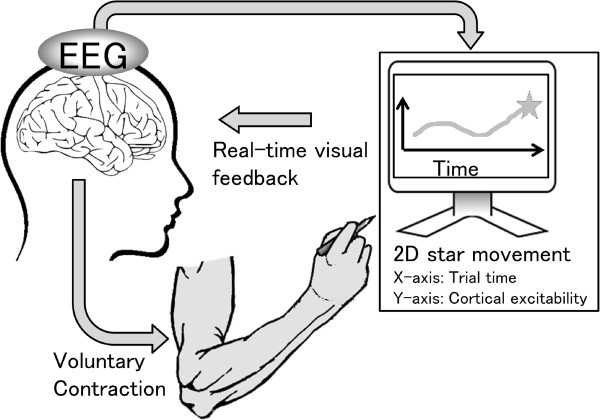
**Schematic diagram of the brain-computer interface that feeds back information about cortical excitability in the sensorimotor cortex.**

To prove its concept, the present study employed a BCI paradigm to provide visual feedback of ongoing EEG features that represents the exaggerated excitability of the sensorimotor cortex during hand movement (Figure 1), and assessed neurological and behavioral changes through 5-month use in a WC patient as a clinical pilot study^a^.

## Results and discussion

**Figure 2:**
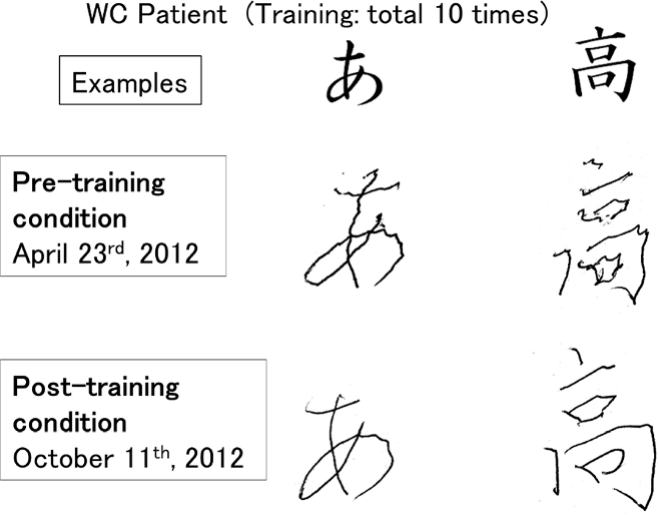
**An example of Japanese and Chinese characters and the handwriting of the patient (Pre-training and post-training condition).**

After 10 BCI training sessions, the WC participant clearly showed a reduction of dystonic movement during writing, and their handwriting of Japanese characters showed a reduction in distortion (Figure 2). The patient also showed some reduction in hand rigidity during writing.

**Figure 3:**
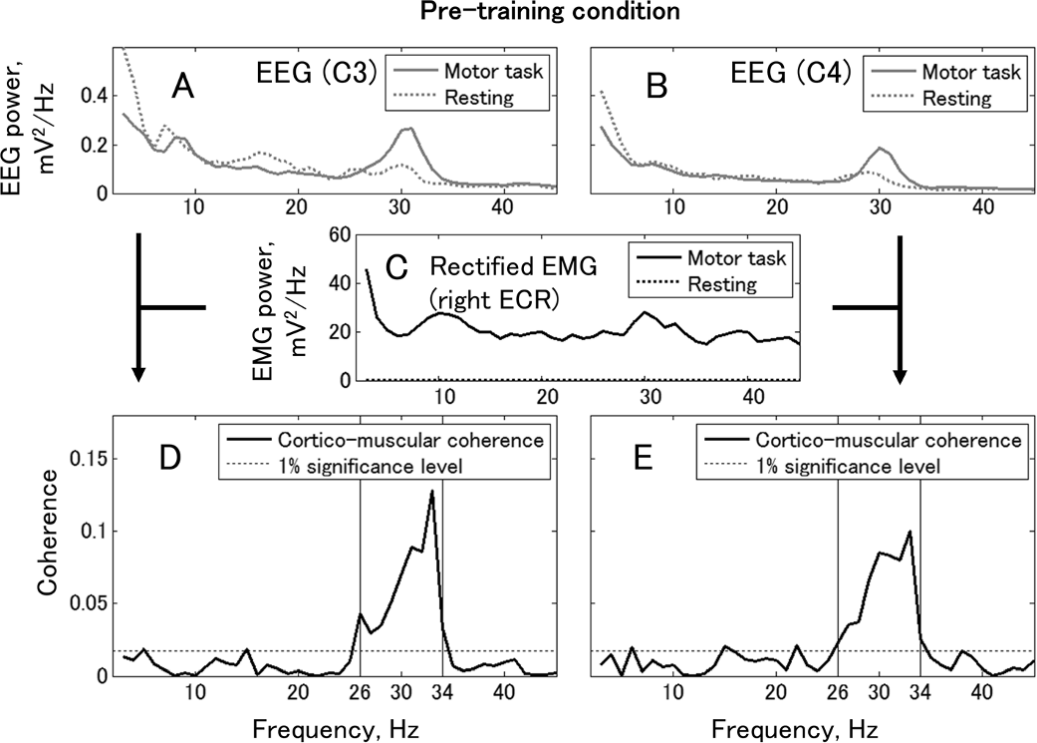
**Cortico-muscular coherence and power spectra of EEG and rectified EMG in pre-training condition.** Top row shows EEG power spectra from the left EEG **(Panel A)** and the right EEG **(Panel B)**. Middle row shows the power spectrum of rectified EMG recorded from the right ECR muscle **(Panel C)**. Bottom row shows coherence between EEG and rectified EMG of the right ECR. The horizontal lines denote the 99% confidence limit. Panels **D** and **E** show the Laplacian EEG from the left and right hemispheres, respectively.

**Figure 4:**
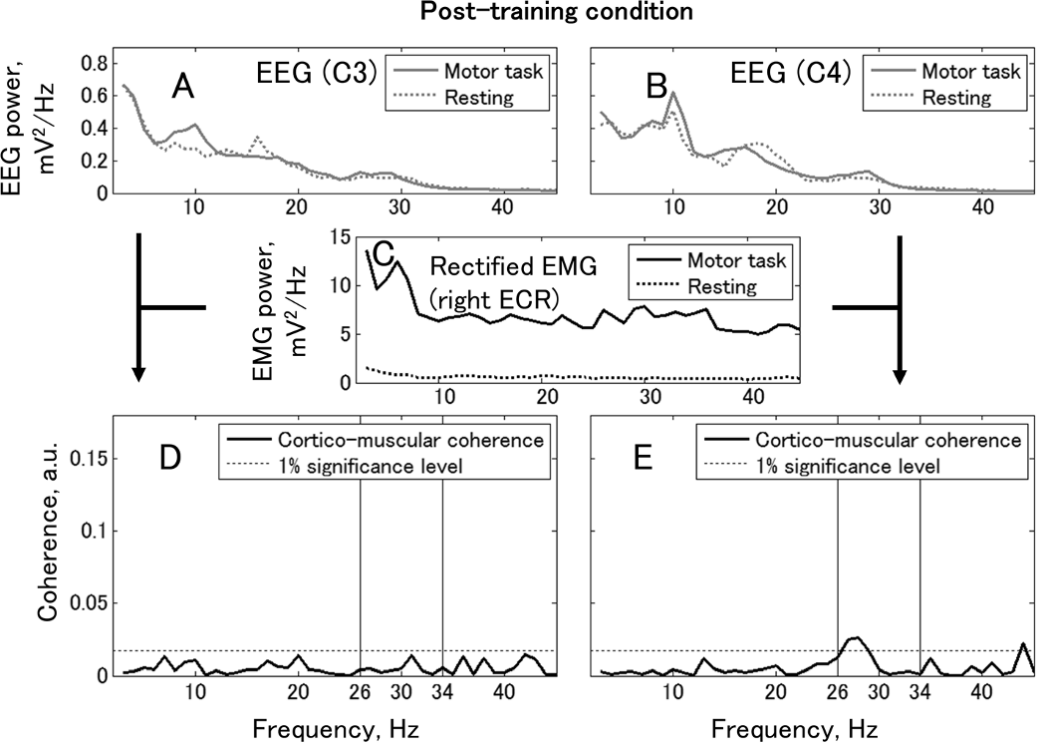
**Cortico-muscular coherence and power spectra of EEG and rectified EMG in post-training condition.** Top row shows the power spectra from the left EEG **(Panel A)** and the right EEG **(Panel B)**. Middle row shows the power spectrum of rectified EMG recorded from the right ECR muscle **(Panel C)**. Bottom row shows coherence between EEG and rectified EMG. The horizontal lines denote the 99% confidence limit. Panels **D** and **E** show EEG from C3 and C4, respectively.

For further analysis, EEG over the bilateral sensorimotor cortex and EMG from the dystonic right extensor carpi radialis (ECR) were recorded during tonic wrist extension, and the power spectra and coherency of the EEG signals and the rectified EMG signal were calculated (Figures 3 and 4). In the pre-training condition (Figure 3), the coherence peak achieved 0.12 between the left EEG and the rectified EMG. A coherence peak was also found between the right EEG and the rectified EMG. The frequency band of significant coherence expanded from 26 Hz to 34 Hz both in the left EEG-EMG coherence (peak value at 33 Hz = 0.12) and in the right EEG-EMG coherence (peak value at 33 Hz = 0.10). In contrast, the post-training condition data showed no significant coherence between the left EEG-EMG, and a low peak (peak value at 28 Hz = 0.03) in right EEG-EMG coherence.

**Figure 5:**
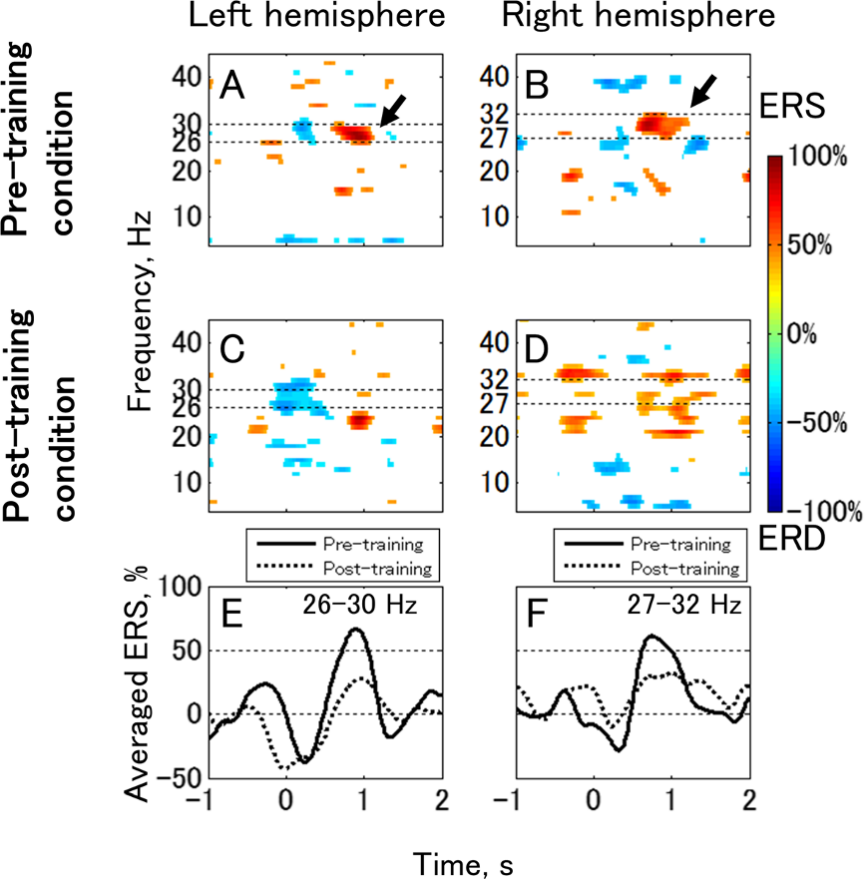
**ERD/ERS produced by repetitive motor task.** Panels **A** and **B** are computed from data recorded prior to training and Panels **C** and **D** are from after training. The lower panels show averaged ERD/ERS values in the 26–30 Hz band **(Panel E)** and in the 27–32 Hz band **(Panel F)** for comparison between pre- and post-training conditions. The left column **(Panels A, C, and E)** and the right column **(Panels B, D, and F)** represent the data recorded from around C3 (left hemisphere) and C4 (right hemisphere), respectively. Statistically non-significant areas are shown as blank in Panels **A–D**. The significance level is set to P < 0.01.

In the ERD/ event-related synchronization (ERS) analysis for EEG data during a repetitive motor task, significant extension-related EEG power increase (ERS) in the beta band was observed bilaterally in the pre-training condition (Figure 5A–B; significance level = 99%). The frequency bands of this ERS expanded from 26 Hz to 30 Hz in the left EEG and from 27 Hz to 32 Hz in the right EEG. Both frequency bands were within the frequency band of dystonia-specific EEG features determined by the coherence analysis. After BCI training, the averaged ERS decreased bilaterally from 67% to 28% in the left EEG (in 26–30 Hz, Figure 5E) and from 62% to 32% in the right EEG (in 27–32 Hz, Figure 5F).

Certainly, there is some evidence for the involvement of the bilateral motor cortices in the abnormal muscle activity in focal dystonia. Tecchio et al. reported patients’ exaggerated and bilaterally coupled cortico-muscular coherence in the beta band (mean frequency 21.5 Hz) during isometric contraction [16], which is not seen in healthy participants (e.g. [17]). These studies suggest the existence of different mechanisms of cortico-muscular coupling from healthy individuals in dystonia patients. We found a large beta ERS from the hand motor areas in both hemispheres in the pre-training condition (Figure 5). Since such EEG characteristics were consistent with previous coherence studies [18], it is natural to assume that large ERS is observed in the frequency in which large cortico-muscular coherence exists. Therefore, we consider that selecting the 26–34 Hz EEG amplitude to represent dystonia-specific EEG features was reasonable.

Cortico-muscular coherence provides a quantitative measure of the linear components of the coupling between EEG and rectified EMG. It should be noted that the coherence is not expected to be a direct estimate of the anatomical neural connections. Tecchio et al. argue that high cortico-muscular coherence found in dystonic patients should be understood in a physiological sense as signs of a different (abnormal) sensorimotor integration [16]. They also suggest that healthy subjects transiently mediate movement control by tuning their neuronal firing rates more than by cortico-muscular synchronization, and that the repertoire of voluntary motor control strategies is reduced in dystonic patients. We agree with this suggestion and the improvement of writing after BCI training (Figure 2) can be explained by a change in the motor control strategy for writing. The cortico-muscular coherence might quantitatively indicate this change from synchronization leading control to other modes, for example, firing rate tuning.

Our result clearly shows a decrease of ERS in the beta frequency band associated with a significant improvement of handwriting after biweekly BCI training. Since the ERS in this beta frequency band must be a dystonia-related feature, providing visual feedback of the ongoing ERS level through our BCI system might promote plastic and functional reorganization in the neural network of this patient. Considering that it has been over 5 years since the appearance of symptoms, and that conventional treatments were not effective for this patient, BCI training might have different applications for functional cortical modulation than previously thought.

In addition, in the current study, clinical scales were not measured before or after the intervention. Instead, the handwriting on a paper was employed, and the authors set the main focus of the study on EEG pattern changes induced by our BCI system in a WC patient. Therefore, degree of the functional improvement of the patient's handwriting is still unclear in this study. As a next step, it is necessary to quantify the functional improvement using, for example, the Writer's Cramp Rating Scale [19] or other dystonia rating scales. To inquire more details regarding functional improvement of hand-writing by trainings, it will need long-term follow-up study. There are still open questions whether more training can change EEG pattern or improve hand writing, or whether the improvement of the handwriting can be sustained after finishing the BCI training.

## Conclusions

To date, mainstream BCI rehabilitation has aimed to induce functional reorganization for stroke patients or incomplete spinal cord injury patients [4]. The current study, however, demonstrates another application of therapeutic BCI for patients with exaggerated cortical activity associated with involuntary muscle contractions, such as a dystonia patient, using visual feedback of abnormal EEG activity. Though this study is a single-case study without control participants and is also in the pilot stage, the encouraging results may facilitate further larger scale studies or randomized controlled trials.

## Methods

The WC patient participated biweekly training for 5 months (10 times) with a BCI system that detects dystonic contraction-related EEG features and displays them for the patient. The EEG features were calculated from bilaterally recorded EEG in 26–34 Hz frequency bands, where significant cortico-muscular coherence was observed during tonic ECR muscle extension in the right (dominant) dystonic hand. During the BCI training, the patient was instructed to repeat hand extension for 70 seconds, and, during extension, keep the amount of dystonia-specific EEG features low level, the same as during resting, using the feedback from the BCI.

### Participant

A patient with the diagnosis of WC (age 67 years; female; 1.5 years from WC onset) participated in this pilot study. The patient was right-handed and showed WC-specific symptoms, such as feeling difficulty in writing but not in other hand motor functions. The diagnosis was provided by a neurologist and physiatrist. Since the WC was not caused by secondary causes, such as drug use or head injury, the patient classified as primary dystonia patient. Moreover the WC in this study was categorized sporadic dystonia same as to general type of WC patients. The patient had been on medication for one year, but treatment was halted since the improvement of symptoms was not confirmed before this study started. Incidentally, the botulinum toxin injection for WC or focal hand dystonia is not covered by health care insurance and not practiced in Japan, so the patient had never received it.

The patient gave written informed consent for this study and the publication of individual data, which was approved by the local ethical committee of Asahikawa Medical University Hospital, and the study was conducted in accordance with the Declaration of Helsinki. The patient participated in the experiment twice a month over a 5-month period, a total of 10 days. Every experiment was finished within 2 hours to avoid fatigue.

### Biosignal measurement

We recorded 10-channel monopolar EEG and 3-channel surface EMG from the forearm during the experiment with Ag/AgCl electrodes (diameter 9 mm). To record scalp EEG, the electrodes were placed at C3 and C4, as designated according to the International 10/20 system, and 2.5 cm anterior, posterior, left, and right to C3 and C4, close to the hand representation area. All EEG channels were referenced to the right ear lobe. The ground electrode was positioned on the forehead. The EEGs were then converted to a reference-free form by a Laplacian algorithm [20] that used the set of the four neighbor electrodes. For electrode C3 (C4), these were anterior, posterior, left, and right to C3 (C4). The two Laplacian EEGs are called the left EEG (C3) and the right EEG (C4) in this study.

The surface EMG of the right hand (dominant hand) was recorded from the ECR muscle.

All biosignals were band-pass filtered between 2 and 1000 Hz, and simultaneously digitized at 2400 Hz using a biosignal amplifier (g.USBamp, gtec, Austria). The EEG data were used online and were also stored on a personal computer for offline analysis.

### Assessment set

In each daily assessment, we conducted EEG and EMG measurement during simple motor tasks both before and after 1-hour BCI training to assess the electrophysiological signal changes.

For each assessment, the patient was requested to perform the following: (1) tonic contraction of the right ECR muscle (tonic motor task) for 70 s; (2) right hand extension with a rhythmic auditory cue 40 times, one every 2.5 s (repetitive motor task); and (3) write Japanese and Chinese characters with a ballpoint pen (writing task).

During the tonic motor task, the participant maintained a constant and weak extension force with integrated EMG recorded from the right ECR muscle. Integrated EMG displayed for the patient was computed, rectifying and smoothing with a moving average with a window of 1 s.

### Time-frequency representation

In the EEG analysis, we drew ERD/ERS maps using the intertrial variance method [21–23]. The two Laplacian EEG data sets from the repetitive motor task were analyzed between 5 and 50 Hz in intervals of 2 Hz. Trials were filtered with a 4th order Butterworth filter, and the ensemble average was subtracted from individual trials (total 40 trials). This operation reduces the contribution of phase-locked responses to the ERD/ERS quantification. The trials were squared and then averaged using a moving average window of 250 ms smoothed to estimate the power change in each frequency band. The reference interval for the relative power changes was between −1.5 and −0.5 s from the onset of the EMG from the right ECR.

The statistical significance of the ERD/ERS values was determined by applying a t-percentile bootstrap algorithm [24] with a significance level of 1%. Significant ERD/ERS data were shown as time-frequency maps.

### Cortico-muscular coherence analysis

To access the linear involvement of cortical oscillations in motor unit synchronization, cortico-muscular coherence analysis was conducted on bilateral EEG and dystonic right EMG during the tonic motor task. The computation process was the same as in previous cortico-muscular coherence studies [25–27]. Following segmentation of the data stream into 70 segments of 1-s duration, the segment was windowed with a 1-s Hamming window (90% overlap), and a Fourier transform was used. The segment length of 1 second leads to a 1-Hz frequency resolution. The 99% confidence limit determined by Monte Carlo simulation [28] was approximately 0.017.

### BCI paradigm

During BCI training, a visual feedback monitor displayed a star as moving upward or downward based on the EEG features-related dystonic muscular contraction with an update rate of 16 Hz. The same visual feedback method was employed in a previous study [8].

Using EEG time-frequency maps, we identified EEG frequency bands that reflected the cortical excitability of the sensorimotor cortex. We also confirmed the influence of the EEG oscillation in these frequency bands on the EMG of the forearm muscles using cortico-muscular coherence analysis. As visual feedback in the BCI intervention, the logarithms of the band power of EEG recorded from the bilateral hemispheres (*x*_1_ and *x*_2_) were used for linear discriminant analysis (LDA) to detect the dystonia-specific power changes as feature values.

The LDA classifier parameters (weights *w*_1_ and *w*_2_ and a constant *c*) were calculated using the data of repetitive motor tasks in pre-BCI neurorehabilitation. The calculation was done based on cue-based 2 class wise classification, the same as a previous study [29], and we picked the LDA parameters that showed the best classification accuracy in 2 cross-fold validation.

**Figure 6:**
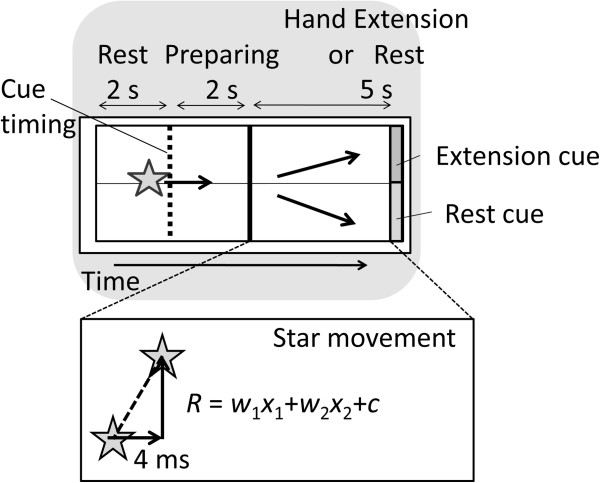
**Visual feedback on the computer display for the WC patient.**



The LDA classification result (*R*) was converted to the online movement of a star in the Y-axis as visual feedback for the patient (Figure 6). The star’s movement was calculated based on the following equation:

The displacement of the star, *R*, *x*_*1*_, and *x*_*2*_ calculated from the last 1-s data were updated every 4 ms (250 Hz) in the PC processing. Due to the refresh rate of the PC screen, the actual refresh rate was approximately 60 Hz. A positive/negative value of *R* was translated into the representation of a right hand extension/resting, and into an upward/downward displacement of the star.

All analyses described above were carried out using MATLAB software (The MathWorks, US) with custom-developed programs.

## Endnote

^a^Short report including preliminary data, which was partly used in this study, nominated as one of top 10 reports for “The International Annual BCI Award,” supported by g.tec, 2013.
